# Thermal Shock Resistance Enhancement of Alumina-Based Castables by Incorporating Porous Alumina Spherical Aggregates

**DOI:** 10.3390/ma19173761

**Published:** 2026-09-04

**Authors:** Tong Fu, Ying Huang, Wei Xiong, Haonan Chen, Qilong Chen, Haoxuan Ma, Quanli Jia

**Affiliations:** 1 Henan Key Laboratory of High Temperature Functional Ceramics, School of Materials Science and Engineering, Zhengzhou University, Zhengzhou 450001, China; 15937801589@163.com (T.F.); chenhaonan2333@163.com (H.C.); 2 Chengdu Futian High Temperature Materials Technology Co., Ltd., Chengdu 611300, China; 15108413877@163.com (Y.H.); shiongwei@126.com (W.X.); chenqilong1976@163.com (Q.C.)

**Keywords:** alumina spherical aggregates, corundum–spinel castables, thermal shock resistance, porous aggregates, purging plug

## Abstract

Chrome-containing corundum–spinel castables are the preferred materials for ladle purging plugs due to their excellent slag resistance, high mechanical strength, and good volume stability. However, their poor thermal shock resistance often leads to transverse cracking or thermal spalling, thereby limiting their service life improvement. Since aggregates typically constitute 70 wt% of castables, modifying the aggregates may be an effective strategy to enhance the thermal shock resistance. To this end, alumina spherical aggregates were prepared via a pan granulation technique using α-Al_2_O_3_ powders as the raw material and a ZnCl_2_ solution as the binder. The effects of the firing temperature on the physical properties and the microstructure of the as-fabricated aggregates were investigated, followed by an evaluation of their impact on castable performance. After firing at 1400 °C for 3 h, the resulting alumina spherical aggregates exhibited a porous interior and a dense exterior structure, with an apparent porosity of 27.21%, a water absorption capacity of 9.38%, and a crushing rate of 51.3%. When these aggregates were incorporated into castables, their apparent porosity increased, while the bulk density and the cold strength showed slight reductions; however, their thermal shock resistance was notably improved. Among the formulations tested, the castable containing 4% of 0.2–0 mm spherical aggregates exhibited the best thermal shock resistance, with a residual strength of 7.96 MPa and a strength retention rate of 27.85%.

## 1. Introduction

External ladle refining is essential for improving molten steel quality and developing new steel grades. Ladle bottom argon purging is a key method for achieving uniform composition and temperature, as well as facilitating inclusion removal [1,2,3]. As the direct functional component of the purging system, plug performance largely determines the refining efficiency and the ladle turnover rate [2,3,4]. Statistics show that premature plug damage increases refractory consumption, risks secondary pollution, and may even interrupt the refining process [4,5]. During the steel-making process, purging plugs endure harsh conditions: repeated thermal shocks from contact with molten steel (>1600 °C) and intermittent argon blowing, alongside high-temperature erosion, slag attack, and mechanical stress [4,5,6]. Moreover, cold argon passing through the ~1600 °C plug core creates a steep temperature gradient within the slit region. These factors collectively cause fracture failure and thermal spalling, reducing the plug service life [7,8,9,10]. To meet these demanding and severe requirements, purging plugs needed to be possessed of higher strength, good thermal shock resistance (labelled as TSR) and slag resistance.

Among Al_2_O_3_-SiO_2_, Al_2_O_3_, Al_2_O_3_-MgO, etc., castables, calcium aluminate cement (labelled as CAC) bonded chrome-containing corundum–spinel materials have become the preferred choice for purging plugs, owing to their high strength, excellent slag corrosion resistance, and good volume stability. These properties are attributed to the formation of Al_2−x_Cr_x_O_3_, Ca_4_Al_4_CrO_16_, and Mg Al_2−x_Cr_x_O_4_ solid solutions [8,9]. Nevertheless, in practice, these materials still suffer from typical failure modes such as thermal spalling, structural spalling, and steel penetration into the slits. However, the Cr_2_O_3_-containing plugs possess worse TSR in comparison with plugs without Cr_2_O_3_, thereby decreasing their service life and gas bubbling ability because of thermal spalling or fracture [9,10,11].

To date, various efforts have been made to improve the TSR of alumina–spinel purging plugs, such as introducing nano-alumina powders, zirconia, or calcium aluminate [12,13,14]. However, poor TSR remains a critical issue limiting any further extension of their service life. Given that aggregates account for up to 70 wt% of castables, modifying them offers a promising approach. Several studies [14,15,16,17,18,19,20] have improved TSR by incorporating different aggregates, including porous alumina, CaO·MgO·Al_2_O_3_, bonite (labelled as CA_6_), zirconia–alumina, resin-coated alumina, and alumina bubbles. Nevertheless, challenges such as high cost and complex procedures remain to be overcome.

Spherical aggregates offer advantages such as good flowability, low water demand, and reduced stress concentration due to their smooth edges, thereby enhancing the thermal shock resistance [21,22,23,24,25]. Thus, we proposed a technique combining a spherical shape with a porous structure, which may enhance the performance of purging plugs. To address these challenges, porous alumina spherical aggregates with a tailored structure were prepared and incorporated into alumina–spinel castables, and their effects on the properties of the castables were investigated in this work.

## 2. Experimental

### 2.1. Preparation of Alumina Spherical Aggregates

**Figure 1:**
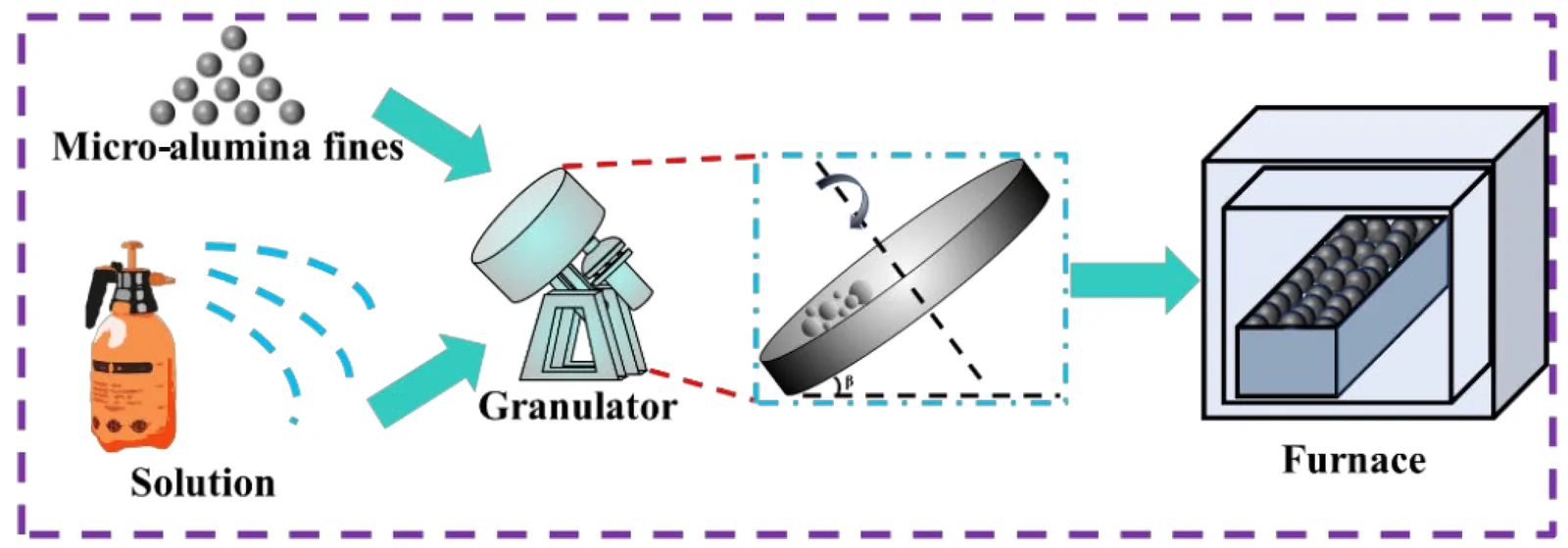
Schematic diagram of procedure for preparing alumina spherical aggregates.

Alumina spherical aggregates (labelled as ASAs) were prepared from α-Al_2_O_3_ powders (99.6%, D_50_ = 5 μm, Macklin Biochemical Technology Co., Ltd., Shanghai, China) using an 8% ZnCl_2_ solution (10 mass%, Henan Tianma New Materials Co., Ltd., Zhengzhou, China) as a binder. After granulation (10 rpm), the spheres were dried at 110 °C for 24 h, sieved into three fractions (1–0.5, 0.5–0.2, and 0.2–0.09 mm), and fired at 1300–1500 °C for 3 h (see Figure 1). The vacuum evacuation method was employed for water absorption of ASAs according to Chinese standard GB/T 2999-2016 [26]. The residual mass ratio of ASAs was determined by reweighing 200 g ASAs after crushing.

### 2.2. Preparation of Alumina–Spinel Castables

Tabular alumina (Al_2_O_3_: 99.20%, 6–3 mm, 3–1 mm, 1–0.09 mm, Almatis Co. Ltd., Qingdao, China), white fused corundum powder (Al_2_O_3_: 99.0%, <0.088 mm, Henan Sicheng Grinding Technology Co., Ltd., Zhengzhou, China), fused spinel powder (<0.044 mm, Kaifeng Tenai Co., Ltd., Kaifeng, China), ultrafine alumina (Al_2_O_3_: 99.10%, D50 = 1.2 µm, Henan Tianma New Material Co., Ltd., Zhengzhou, China), chromium oxide powder (Cr_2_O_3_ > 98.0%, <20 µm, Henan Huaxian Jinlong Chemical Plant, Anyang, China), CAC (Secar 71, Imerys China Co., Ltd., Beijing, China), and ZnCl_2_ fines (AR, <0.044 mm, Macklin Biochemical Technology Co., Ltd., Shanghai, China) were applied as starting materials. The dispersant was 0.15% Castment FS 10 (BASF SE, Ludwigshafen, Germany). Effects of ASA particle size (1–0.5 mm, 0.5–0.2 mm, and 0.2–0 mm) and addition level (0, 2, 4, and 6 wt%) on the properties of the castables were investigated. The aggregate-to-matrix mass ratio was fixed at 70:30. The matrix was comprised of 12% fused white corundum, 8% fused spinel, 6% α- Al_2_O_3_ ultrafine powder, 2% Cr_2_O_3_, and 2% calcium aluminate cement. After weighing and mixing, water was added to prepare the castables, which were then cast into specimens of 150 × 25 × 25 mm. Following curing at room temperature and demolding, the samples were dried at 110 °C for 24 h and subsequently fired at 1100 °C, 1400 °C, and 1600 °C for 3 h.

Physical properties of the castables fired at 1100 °C, 1400 °C, and 1600 °C for 3 h, including bulk density (labelled as BD), apparent porosity (labelled as AP), cold modulus of rupture (labelled as CMOR), cold crushing strength (labelled as CCS), and permanent linear change (labelled as PLC), were measured according to Chinese standards (GB/T 5072-2023 [27], GB/T 3001-2017 [28], GB/T 2997-2015 [29], GB/T 5988-2022 [30] and GB/T 3000-2016 [31]). The hot modulus of rupture (labelled as HMOR) of the fired samples was measured at 1400 °C for 0.5 h. Thermal shock resistance (labelled as TSR) was evaluated via air-cooling (ΔT = 1100 °C, 3 cycles), and residual strength (labelled as CMOR_at_) and residual strength ratio (labelled as RSR) were used to evaluate their TSR. Phase composition was analyzed by X-ray diffraction (XRD), while microstructures and cracks were observed using laser microscopy (OLS4100, Olympus, Tokyo, Japan), scanning electron microscopy (SEM, ZEISS EVO HD15; ZEISS, Oberkochen, Germany) with energy dispersive spectroscopy (EDS) and laser microscopy.

## 3. Results and Discussion

### 3.1. Properties of Alumina Spherical Aggregates

**Figure 2:**
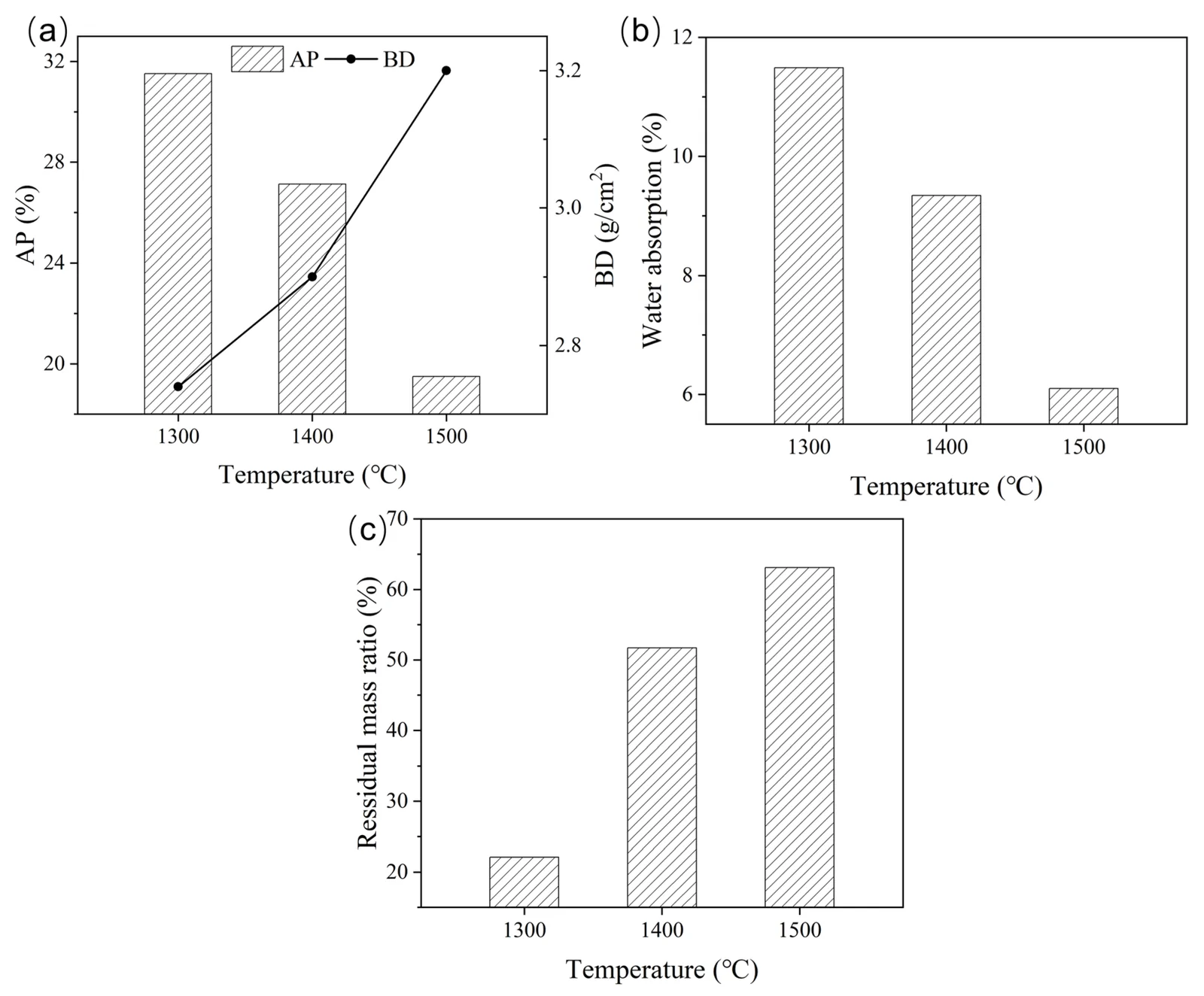
Effect of firing temperature on the properties of the samples: (**a**) AP, BD, (**b**) water absorption, and (**c**) residual mass ratio.

Figure 2 presents the AP, the BD, the water absorption and the crushing rate of the spherical aggregates as a function of the firing temperature. As the temperature increases from 1300 °C to 1500 °C, the AP value decreases from 31.5% to 19.5%, while the BD value increases from 2.74 g/cm^3^ to 3.20 g/cm^3^ (Figure 2a). Concurrently, the water absorption capacity declines from 11.5% to 6.1% (Figure 2b). The residual mass rate of the ASAs after crushing rises significantly from 22.0% to 63.1% (Figure 2c), indicating a significant gain in their strength. However, excessive strength usually indicates a denser and more brittle structure, which can lower the fracture toughness and thus degrade the TSR. Therefore, a firing temperature of 1400 °C for 3 h was selected. The BD of the ASAs after firing at 1400 °C is 2.9 g/cm^3^, much lower than that of tabular alumina aggregates (about 3.58 g/cm^3^), indicating that the volume ratio of the fixed amount of the ASAs is much higher than that of tabular alumina. It will harm their strength but improve their TSR.

Pore formation in the ASAs arises from two sources: (1) moisture evaporation from the solution introduced during granulation, and (2) packing gaps between the α-Al_2_O_3_ particles during granulation. ZnCl_2_ influences the distribution, morphology, and size of these pores. As the firing temperature increases, the alumina particles bond more tightly, the internal gases are expelled, and both the apparent porosity and the water absorption capacity decrease.

**Figure 3:**
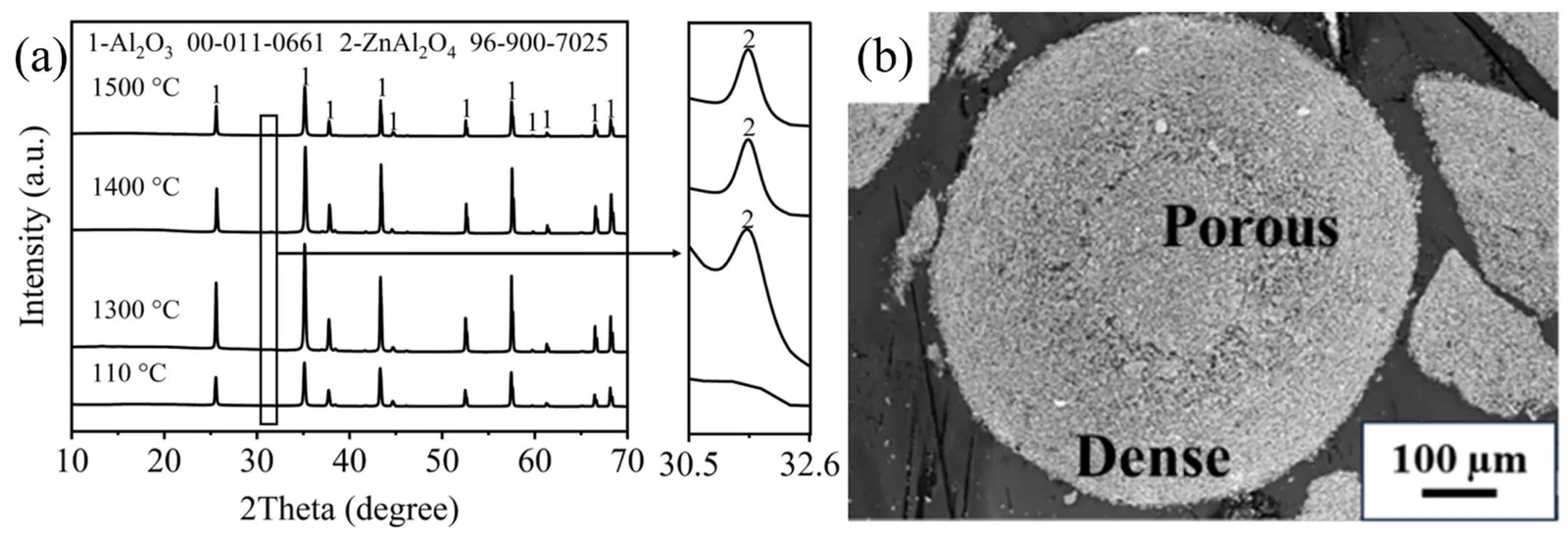
(**a**) XRD patterns of ASAs fired at different temperatures and (**b**) SEM image of an ASAs.

The XRD and SEM analyses were conducted to investigate the phase composition and the microstructure of the 1–0.5 mm ASAs (Figure 3). As shown in Figure 3a, increasing the heat-treatment temperature does not significantly alter the phase composition. The primary crystalline phase remains corundum (ICDD No. 00-011-0661), along with a small amount of ZnAl_2_O_4_. This can be explained as follows. During drying, Zn(OH)_2_ forms via Reaction (1). Upon heating to 500 °C, Zn(OH)_2_ decomposes into ZnO and water vapor (Reaction (2)). Above 600 °C, ZnO can react with Al_2_O_3_ to form ZnAl_2_O_4_ (Reaction (3)).

(1) ZnCl_2_ + 2H_2_O → Zn(OH)_2_ + 2HCl

(2) Zn(OH)_2_ → ZnO + 2H_2_O

(3) ZnO + Al_2_O_3_ → ZnAl_2_O_4_

Figure 3b presents an SEM image of a spherical aggregate fired at 1400 °C. The central part appears relatively loose, while the outer layer is denser. This core–shell structure arises from the decomposition of ZnCl_2_, which generates ZnO and HCl. ZnO reacts with Al_2_O_3_ to form ZnAl_2_O_4_, promoting interparticle bonding. Molten ZnCl_2_ (low melting point, about 290 °C) forms a liquid phase that facilitates particle adhesion and tends to volatilize or migrate toward the outer shell, densifying it. Meanwhile, gas volatilization and additive outflow leave the interior porous and loose. The dense outer layer reduces water absorption and demand during castable preparation, helping maintain its strength. In contrast, the loose interior buffers thermal shock via pores, thereby enhancing the castable’s TSR.

### 3.2. Effect of ASAs Addition on the Properties of the Castables

**Figure 4:**
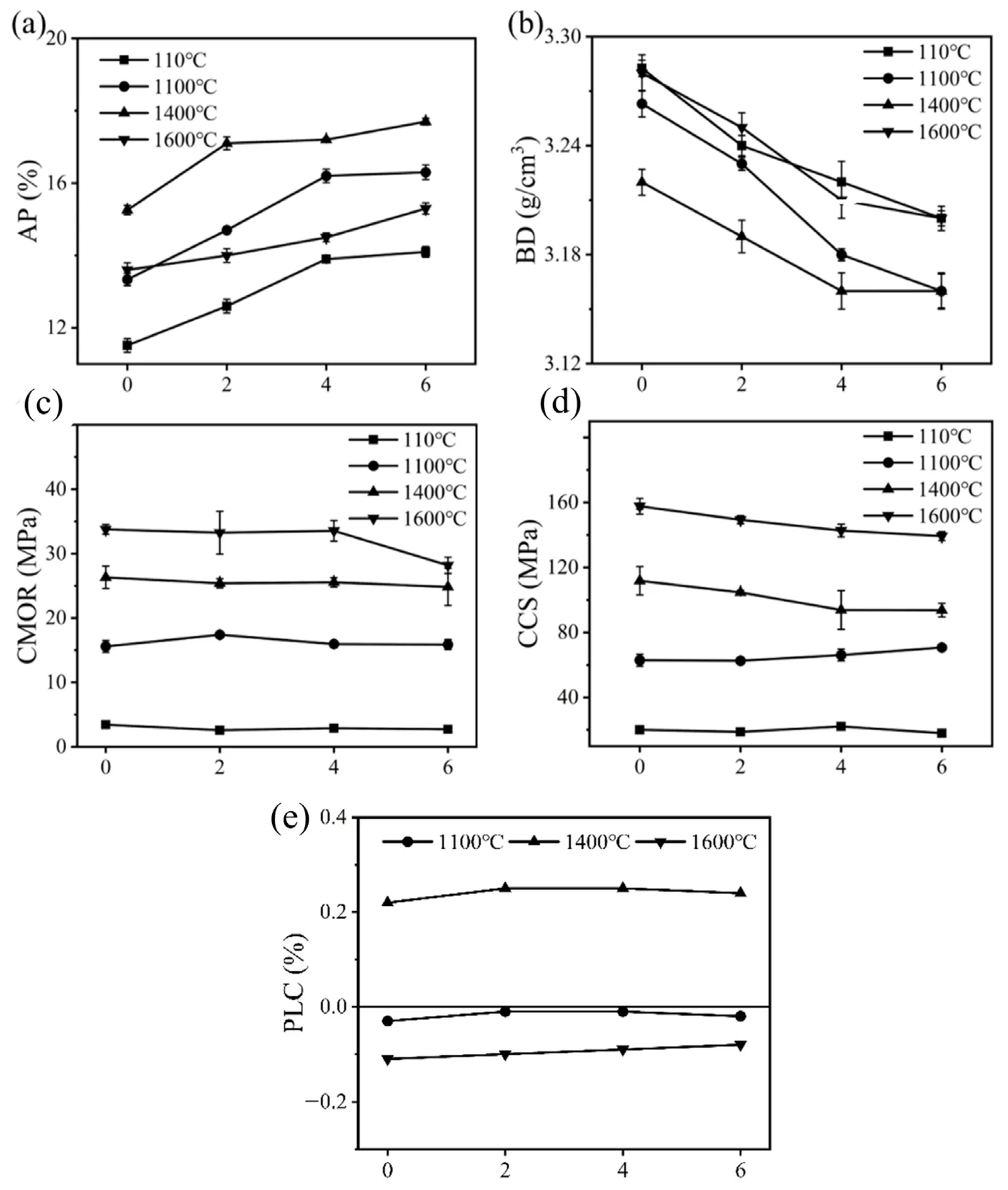
Effect of ASAs content on the physical properties of the castables: (**a**) AP, (**b**) BD, (**c**) CMOR, (**d**) CCS and (**e**) PLC.

Figure 4 illustrates the effects of adding 1–0.5 mm ASAs on the physical properties of the castables. As the ASA content increases, the AP of both the dried and fired samples gradually increases while the BD decreases (Figure 4a,b). Among the samples fired at different temperatures, those sintered at 1400 °C exhibit the highest AP. Specifically, as the ASAs content rises from 0 to 6 wt%, the AP increases from 15.3% to 17.7% and the BD decreases from 3.22 g/cm^3^ to 3.16 g/cm^3^. This trend is attributed to the inherently higher porosity and lower density of the ASAs compared to that of the sample with tabular alumina aggregates. As shown in Figure 4c,d, the strength of the castables increases with ASAs content when fired at 1100 °C but decreases after firing at 1400 °C and 1600 °C. This reduction is primarily attributed to the ASAs’ porous structure, which lowers the particle strength compared to dense tabular alumina aggregates, and its spherical morphology, which reduces the effective load-bearing cross-section and increases local stress. Nevertheless, the overall strength loss remains modest due to the ASAs’ inherent strength and stress-buffering capability. Furthermore, the permanent linear change of the castables shows little variation with ASAs content at all firing temperatures (Figure 4e).

**Figure 5:**
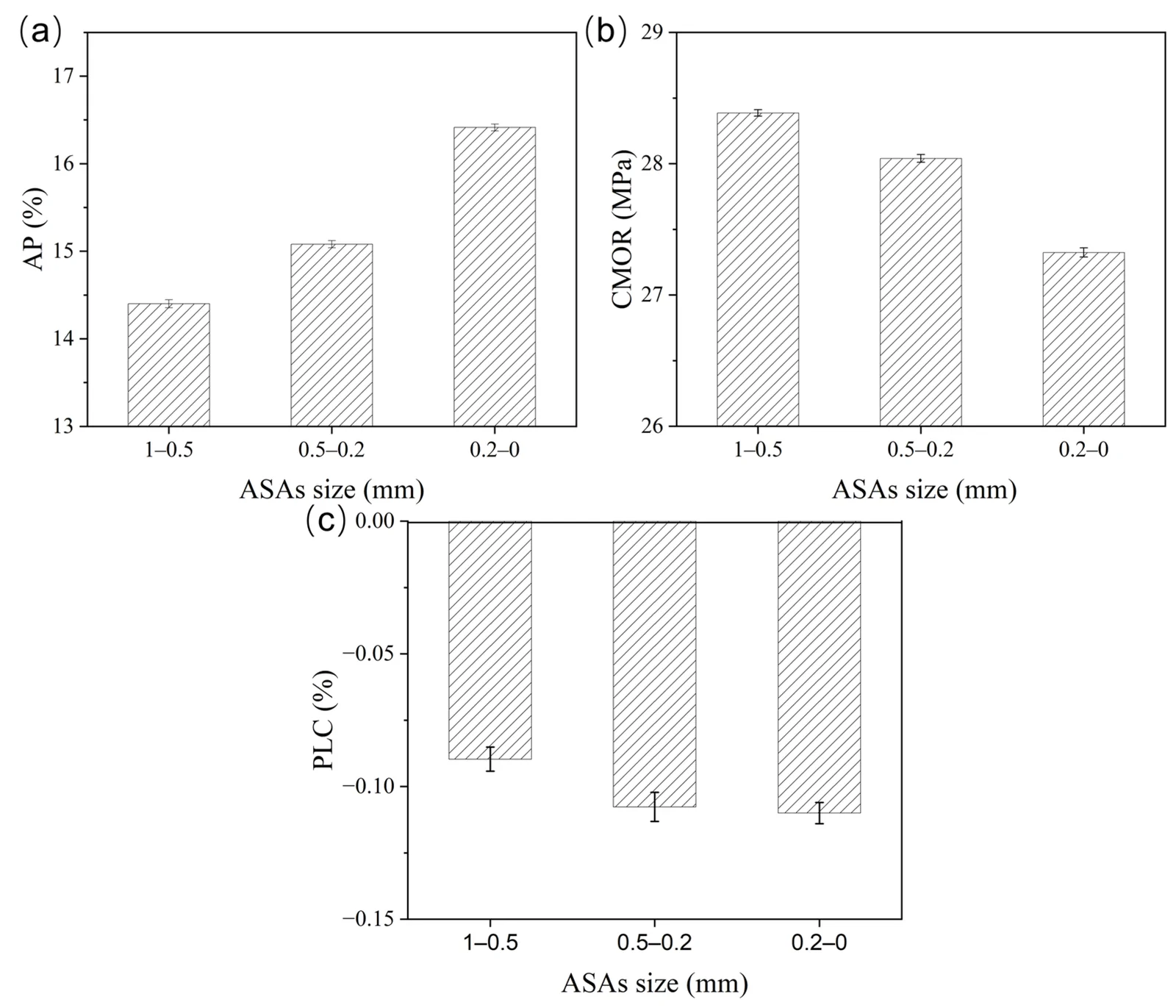
Comparison of (**a**) AP, (**b**) CMOR, and (**c**) PLC of samples with 4% ASAs after firing at 1600 °C.

Figure 5 presents the variations in the AP, the CMOR, and the PLC of the castables containing 4% ASAs of different particle sizes after sintering. As shown in Figure 5a, the AP value gradually increases from 14.36% to 16.37% with decreasing particle size of the ASAs. Conversely, the change in the CMOR follows the opposite trend: as the ASAs particle size decreases, the cold strength slightly declines from 28.34 MPa to 27.32 MPa (Figure 5b). From Figure 5c, it can be observed that the linear shrinkage increases slightly with decreasing ASAs particle size. The samples with 0.2–0 mm ASAs addition possess a higher AP and a lower CMOR.

**Figure 6:**
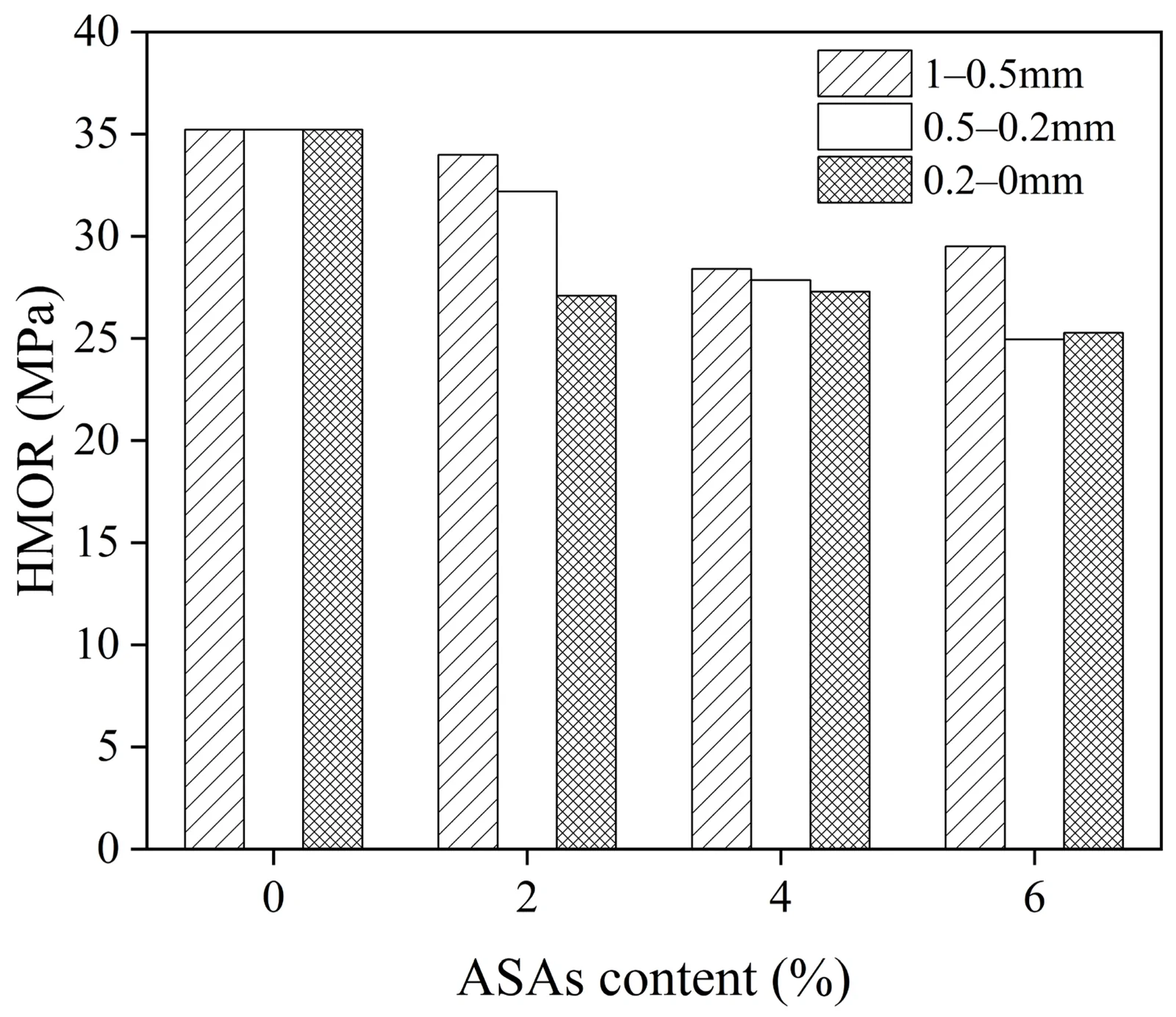
HMOR of samples with different particle size and content of ASAs.

Figure 6 illustrates the effect of the ASAs with different particle sizes on the HMOR of the castables. As the ASAs content increases from 0% to 6%, the HMOR values gradually decrease, which is slightly decreased with the smaller sizes of ASAs particles. Notably, the samples containing 1–0.5 mm ASAs exhibit a higher HMOR value than those with smaller ASAs. Nevertheless, all the HMOR values of the 4% samples with different sizes of ASAs exceed 24 MPa, indicating that the castables with ASAs added still possess a relatively high hot strength, which is similar to the alumina–spinel castables without Cr_2_O_3_ addition [24]. A higher hot strength of the plugs is beneficial for the erosion resistance of the purging plug under service conditions, making their service life longer [24].

**Figure 7:**
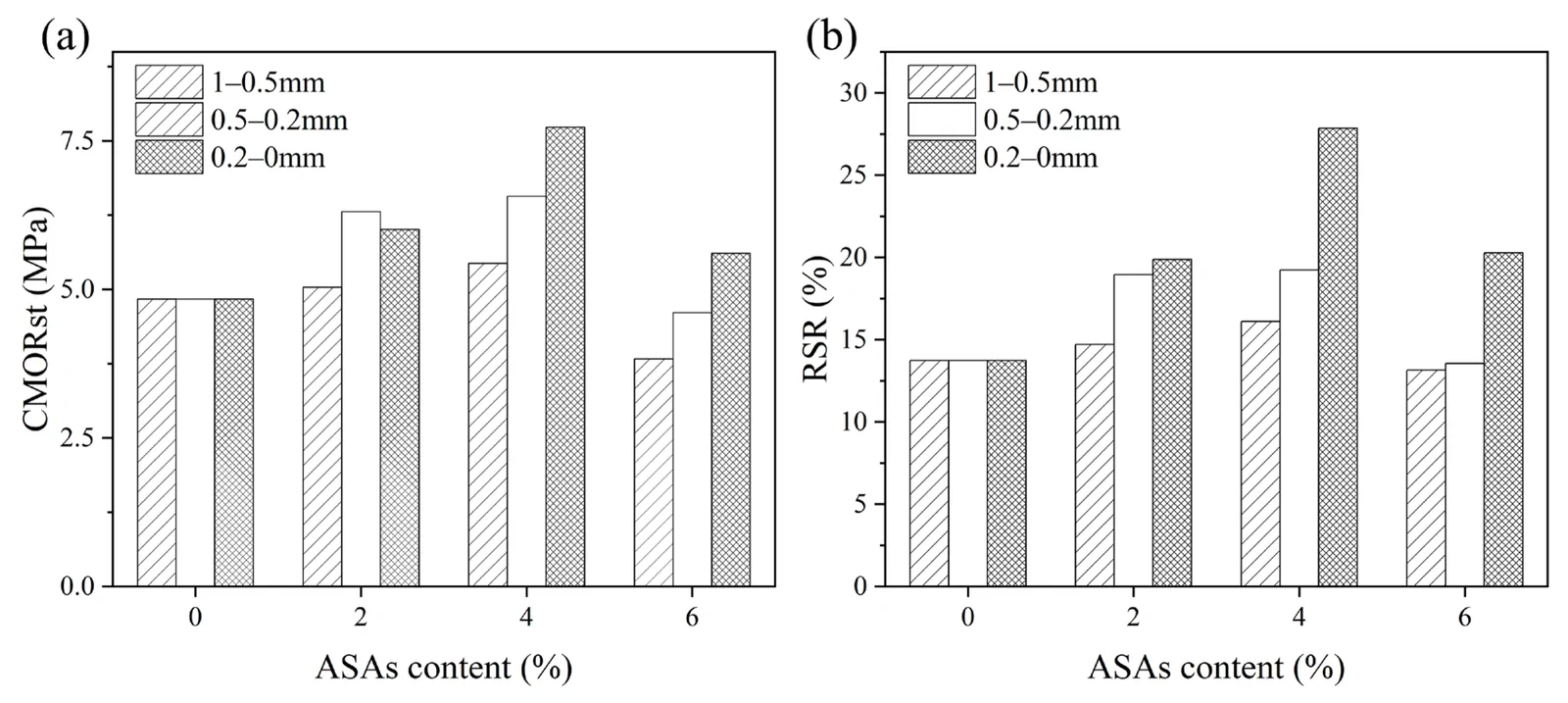
Comparison of (**a**) residual CMOR and (**b**) RSR of samples with different particle sizes and ASAs contents.

Figure 7 illustrates the effect of the content and grain size of the ASAs on the TSR of the castables. As shown in Figure 7, both the residual strength and the RSR gradually increase as the ASAs content rises from 0 to 4%, but they decrease when the content further increases to 6%. The optimal addition level is 4%, achieving residual strengths of 5.40 MPa, 6.31 MPa, and 7.95 MPa, and RSR values of 16.10%, 19.24%, and 27.85% for the different particle sizes. Moreover, a smaller ASAs particle size leads to a better TSR, with the best performance observed in the samples with 0.2–0 mm ASAs. This may be attributed to the larger number and more uniform distribution of the smaller particles within the castables, as well as their higher porosity.

**Figure 8:**
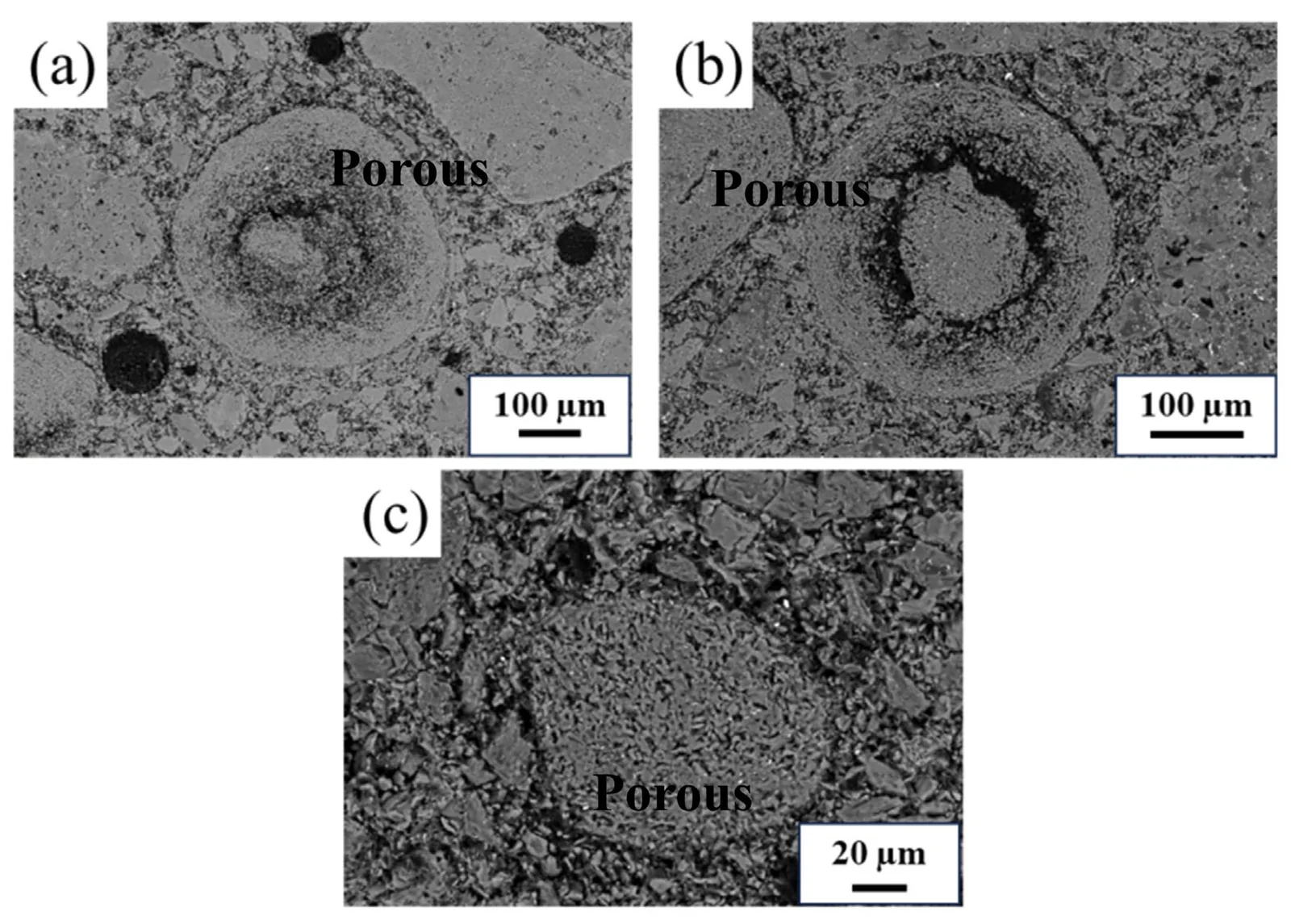
SEM photos of the samples containing 4% ASAs with particle size (**a**) 1–0.5 mm, (**b**) 0.5–0.2 mm, and (**c**) 0.2–0 mm.

To better understand the effect of the ASAs particle size on the TSR, an SEM microstructural analysis was performed on the castables containing ASAs (Figure 8). As presented in Figure 8, the spherical aggregates exhibit a loose interior and a dense exterior, a structure that buffers thermal stress via internal pores while maintaining relatively higher strength, thereby enhancing the TSR. Large particles, formed by the accumulation of smaller nucleated granules during granulation, contain larger interparticle pores that provide limited TSR improvement. In contrast, small particles feature micropores derived from micropowder stacking, thus they more effectively enhance the TSR of the specimens [25,32].

**Figure 9:**
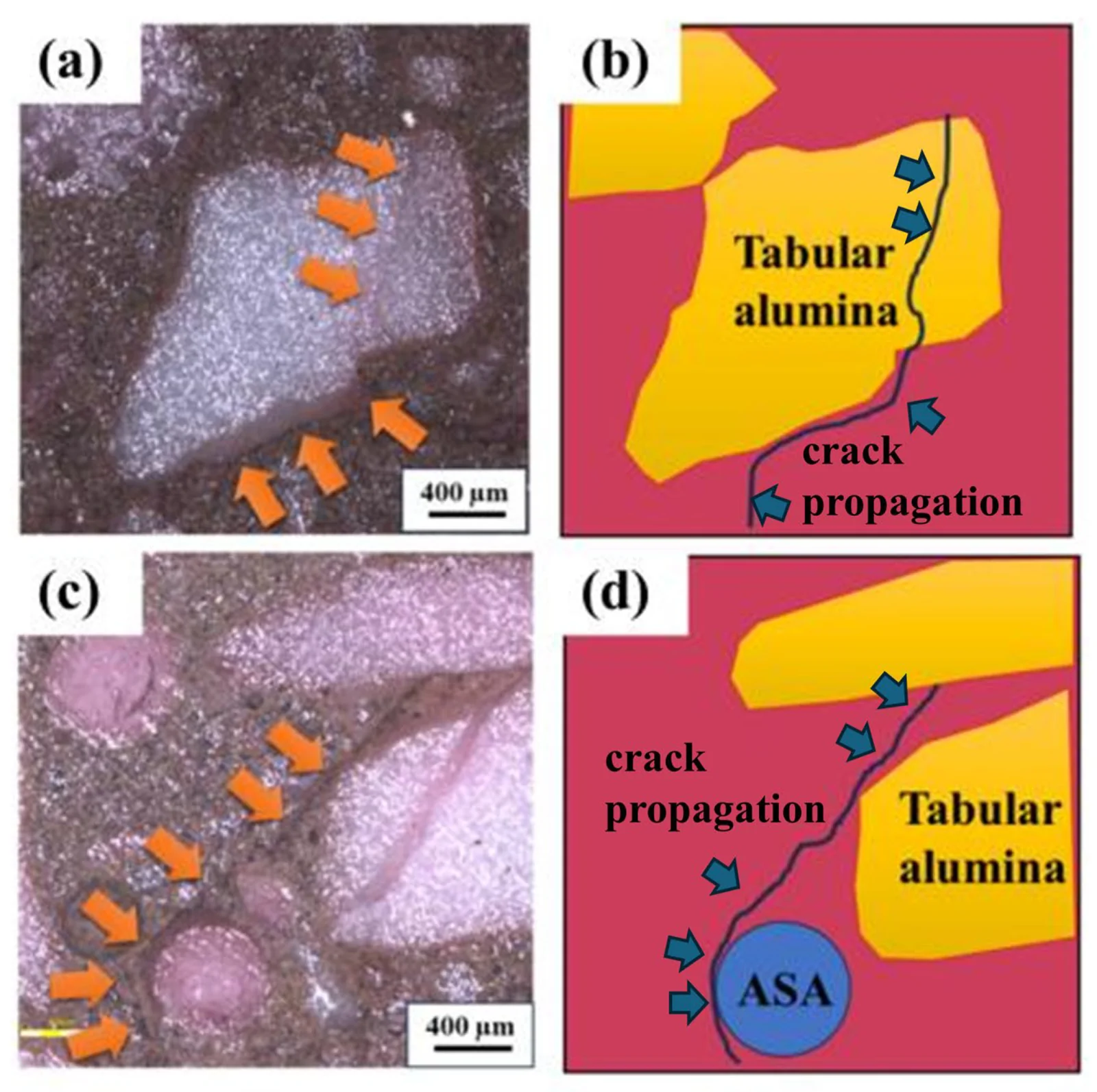
Laser microscope photo and schematic diagram of crack propagation in the samples after thermal shock (**a**,**b**) without ASAs [20]; (**c**,**d**) with 4% 1–0.5 mm ASAs.

The laser microscope images and a schematic crack diagram of the sample containing 4% 1–0.5 mm ASAs after thermal shock are depicted in Figure 9. The Figure shows the reference sample containing tabular alumina aggregates, where the dense and angular tabular alumina particles are evenly distributed within the matrix. After thermal shock, large cracks propagating through the corundum particles are visible, leading to a notable reduction in material strength (Figure 9a,b). Large cracks are observed to propagate along the corundum particles (Figure 9c,d), increasing the crack path length and reducing damage to the material, thereby improving their TSR [18,20]. The reason can be ascribed to the distinct core–shell structure that can be observed in the ASAs particles, characterized by a relatively loose interior and a dense exterior.

The introduction of ASAs into alumina–spinel castables leads to notable changes in their physical and mechanical properties. Regarding their physical properties, the addition of ASAs gradually increases the AP and decreases the BD of the castables, regardless of the firing temperature of the ASAs. This trend is primarily attributed to the inherently higher porosity and lower density of the porous ASAs structure compared to the conventional dense tabular alumina aggregates. Consequently, the cold strength and the hot strength show a slight decline with increasing ASAs content. Despite this decrease, all the HMOR values exceed 24 MPa (Figure 6), indicating that the ASA-containing castables retain a rather higher hot strength. Notably, the samples containing 1–0.5 mm ASAs exhibit a relatively higher HMOR compared to those with finer ASAs particles. Regarding the TSR, the incorporation of ASAs significantly improves both the residual strength and the RSR of the castables. The optimal addition level is 4%, at which the highest TSR is achieved. Furthermore, smaller ASA particles (0.2–0 mm) yield a better TSR than larger ones. This improvement can be explained by the unique loose interior/dense exterior structure of the ASAs (Figure 3b and Figure 8), which buffers thermal stress through internal pores and deflects propagating cracks along the particle edges, extending the crack path and dissipating the thermal shock energy (Figure 9) [16,18,20]. Smaller particles are more uniformly distributed and present in greater numbers at the same addition level, further enhancing this toughening effect.

The enhanced TSR of the alumina–spinel castables containing porous spherical aggregates can be attributed to a synergistic combination of microstructural features. The core–shell structure of the porous spherical aggregates, comprising a porous interior and a dense exterior, modifies crack propagation through crack deflection, branching, and arrest at the shell–core interface. The porous interior dissipates fracture energy via microcracking and local deformation, effectively blunting the crack tip. Additionally, the pores within the aggregate interior accommodate thermal expansion mismatch stress, reducing the thermal expansion coefficient and inducing crack deflection. The spherical morphology further contributes by minimizing the stress concentration at the aggregate corners. Collectively, these mechanisms increase crack path tortuosity, dissipate thermal stress energy, and prevent catastrophic crack propagation, thereby improving thermal shock resistance.

The experimental results show that the ASAs offer a superior TSR: the 4% ASAs (0.2–0 mm) castable achieves a residual strength ratio (RSR) of 27.85%, outperforming alumina bubbles (16.36% [20]). Moreover, the simple pan granulation process makes ASAs a cost-effective alternative for industrial purging plug applications.

## 4. Conclusions

Alumina spherical aggregates were fabricated using ultrafine alumina as the starting material and a ZnCl_2_ solution as the binder, and the effects of the ASAs particle size (1–0.5 mm, 0.5–0.2 mm, and 0.2–0 mm) and addition amount (0, 2%, 4%, and 6%) on the properties of the castables were examined. The main findings are as follows. As the firing temperature increases, the apparent porosity of the alumina spherical aggregates decreases, while their bulk density and crushing rate increase. The resultant alumina spherical aggregates exhibit a flexible buffering structure with a loose interior and a dense exterior. When adding ASAs in the castables, upon increasing the ASAs content from 0% to 6%, it gradually raises the apparent porosity of the specimens and slightly reduces both the cold and hot strength. However, the residual strength and the residual strength ratio after thermal shock improve, reaching optimal levels at 4% ASAs addition. Among the tested particle sizes, the 0.2–0 mm spherical aggregate yields the best thermal shock resistance, which can be ascribed to the core–shell structure of the porous spherical aggregates, comprising a porous interior and a dense exterior, which modifies crack propagation through crack deflection and arrest at the shell–core interface.

## Data Availability

The original contributions presented in this study are included in the article. Further inquiries can be directed to the corresponding authors.
